# The Association of Low-Carbohydrate Diet and *HECTD4* rs11066280 Polymorphism with Risk of Colorectal Cancer: A Case-Control Study in Korea

**DOI:** 10.1016/j.cdnut.2024.102127

**Published:** 2024-02-27

**Authors:** Tao Thi Tran, Madhawa Gunathilake, Jeonghee Lee, Jae Hwan Oh, Hee Jin Chang, Dae Kyung Sohn, Aesun Shin, Jeongseon Kim

**Affiliations:** 1 Department of Cancer AI & Digital Health, Graduate School of Cancer Science and Policy, Goyang-si, Gyeonggi-do, South Korea; 2 Faculty of Public Health, University of Medicine and Pharmacy, Hue University, Hue City, Vietnam; 3 Center for Colorectal Cancer, National Cancer Center Hospital, National Cancer Center, Goyang-si, Gyeonggi-do, South Korea; 4 Department of Preventive Medicine, Seoul National University College of Medicine, Jongno-gu, Seoul, South Korea

**Keywords:** low-carbohydrate diet, *HECTD4* rs11066280, colorectal cancer, case-control study, Korea

## Abstract

**Background:**

Glucose is a main source of energy for tumor cells. Thus, a low-carbohydrate diet (LCD) is thought to make a significant contribution to cancer prevention. In addition, LCD and HECT domain E3 ubiquitin protein ligase 4 (HECTD4) gene may be related to insulin resistance.

**Objectives:**

We explored whether LCD score and HECTD4 rs11066280 are etiological factors for colorectal cancer (CRC) and whether LCD score interacts with HECTD4 rs11066280 to modify CRC risk.

**Methods:**

We included 1457 controls and 1062 cases in a case-control study. The LCD score was computed based on the proportion of energy obtained from carbohydrate, protein, and fat, as determined by a semiquantitative food frequency questionnaire. We used unconditional logistic regression models to explore the association of HECTD4 with CRC prevention and interaction of LCD score and HECTD4 polymorphism with CRC preventability.

**Results:**

Individuals with AA/AT genotypes who carried a minor allele (A) of *HECTD4* rs11066280 exhibited a decreased CRC risk [odds ratio (OR) = 0.75, 95% confidence interval (CI): 0.62, 0.91]. In addition, a protective effect of high LCD score against CRC development was identified (OR = 0.52, 95% CI: 0.40, 0.68, *P* for trend <0.001). However, the effect of LCD depended on individual’s genetic background, which appears only in participants with TT genotype of *HECTD4* rs11066280 [OR = 0.49 (0.36–0.68), *P* interaction = 0.044].

**Conclusions:**

Our findings suggest a protective effect of LCD and a minor allele of *HECTD4* rs11066280 against CRC development. In addition, we provide an understanding of the interaction effect of LCD and *HECTD4* rs11066280 on CRC, which may be helpful for establishing diet plans regarding cancer prevention.

## Introduction

Colorectal cancer (CRC) has been recognized as one of the most frequent cancers with 1.9 million new cases and 935,000 deaths in 2020. It ranks third and second with regard to cancer incidence and mortality, respectively [1]. There is a geographical difference in distributing CRC globally. Asian countries have the highest proportion of incidence and mortality worldwide, accounting for 51.8% and 52.4%, respectively. Notably, these rates have been on the rise in recent years [2]. CRC can be considered a marker of socioeconomic development because a higher incidence rate tends to be observed in countries with high Human Development Index [1]. This trend is no exception in South Korea, where CRC is a major public health concern with an age-adjusted incidence of 28.7 per 100,000 in 2019 [3].

There are several risk factors leading to CRC, including nonmodifiable and modifiable factors [4]. Overweight, obesity, and type II diabetes have been demonstrated to be well-established etiologic factors for CRC linked to insulin resistance [5]. In addition, the influence of macronutrients on CRC has been documented, with high carbohydrate intake considered as an etiological factor because of its impact on insulin secretion [5,6]. Notably, when the intake of one macronutrient increases, the levels of the other macronutrients decrease. To date, it is suggested to assess the overall diet or macronutrient by considering the collective effect of 3 main macronutrients, including carbohydrate, fat, and protein, rather than focusing solely on a single macronutrient. Thus, a simple score called a low-carbohydrate diet (LCD) is created based on the energy percentage from carbohydrate, protein, and fat [[7], [8], [9]].

Glucose is considered the main source of energy for tumor cells. Therefore, LCD is hypothesized to make a significant contribution to cancer prevention [10]. However, the existing literature on the association of LCD with cancer does not seem consistent. For example, a prospective cohort study of 90,171 participants indicated a significantly elevated overall cancer risk as overall LCD score increased. Conversely, participants with higher LCD score exhibited a lower risk of gastric cancer [8]. However, low carbohydrate and high protein score was found to have a null association with overall cancer risk in another prospective cohort study, whereas an inverse association with CRC in females with high saturated fat consumption and a positive association in males with the score from vegetable protein were observed [11]. These ambiguous results raise questions about the role of LCD in carcinogenesis.

Furthermore, genetic polymorphisms are potential contributing factors to the development and progression of CRC. CRC development is a multistep and complex process that involves chromosomal instability or microsatellite instability or proto-oncogenes, tumor-suppressor genes, and epigenetic changes [12,13]. The HECT domain E3 ubiquitin protein ligase 4 (*HECTD4)* gene, also known as *C12orf51*, is located on chromosome 12q24. It may encode E3 ubiquitin protein ligase and involve in protein modification or ubiquitination [14,15]. In addition, genetic variations at 12q24 were reported in relation to cancers [15]. The single-nucleotide polymorphism (SNP) annotated as rs11066280 is a common variant at an intron of the *HECTD4* gene found in the Korean population. Importantly, previous studies have emphasized the potential effect of *HECTD4* rs11066280 on type 2 diabetes and hypertension among the Korean population [[16], [17], [18]]. It is important to note that type 2 diabetes has been widely recognized as a risk factor for CRC development [[19], [20], [21]] because of the underlying mechanisms such as hyperglycemia and insulin resistance [22,23]. However, the association of *HECTD4* rs11066280 with CRC risk has not been investigated so far. In this regard, LCDs, which can impact insulin levels, have been proposed for cancer treatment [24]. Furthermore, the interaction between genes and diet may explain the wide differences in cancer susceptibility across populations [25]. Thus, we proposed a hypothesis on the interaction effect between LCD score and *HECTD4* rs11066280 on CRC risk.

To the best of our knowledge, little is known about the association of LCD with CRC. In addition, the contribution of *HECTD4* rs11066280 to the etiology of CRC has not been reported so far. Thus, our study aimed to elucidate whether LCD score and *HECTD4* rs11066280 are etiological factors for CRC and whether LCD score interacts with *HECTD4* rs11066280 to modify CRC risk.

## Methods

### Study design and participants

**FIGURE 1:**
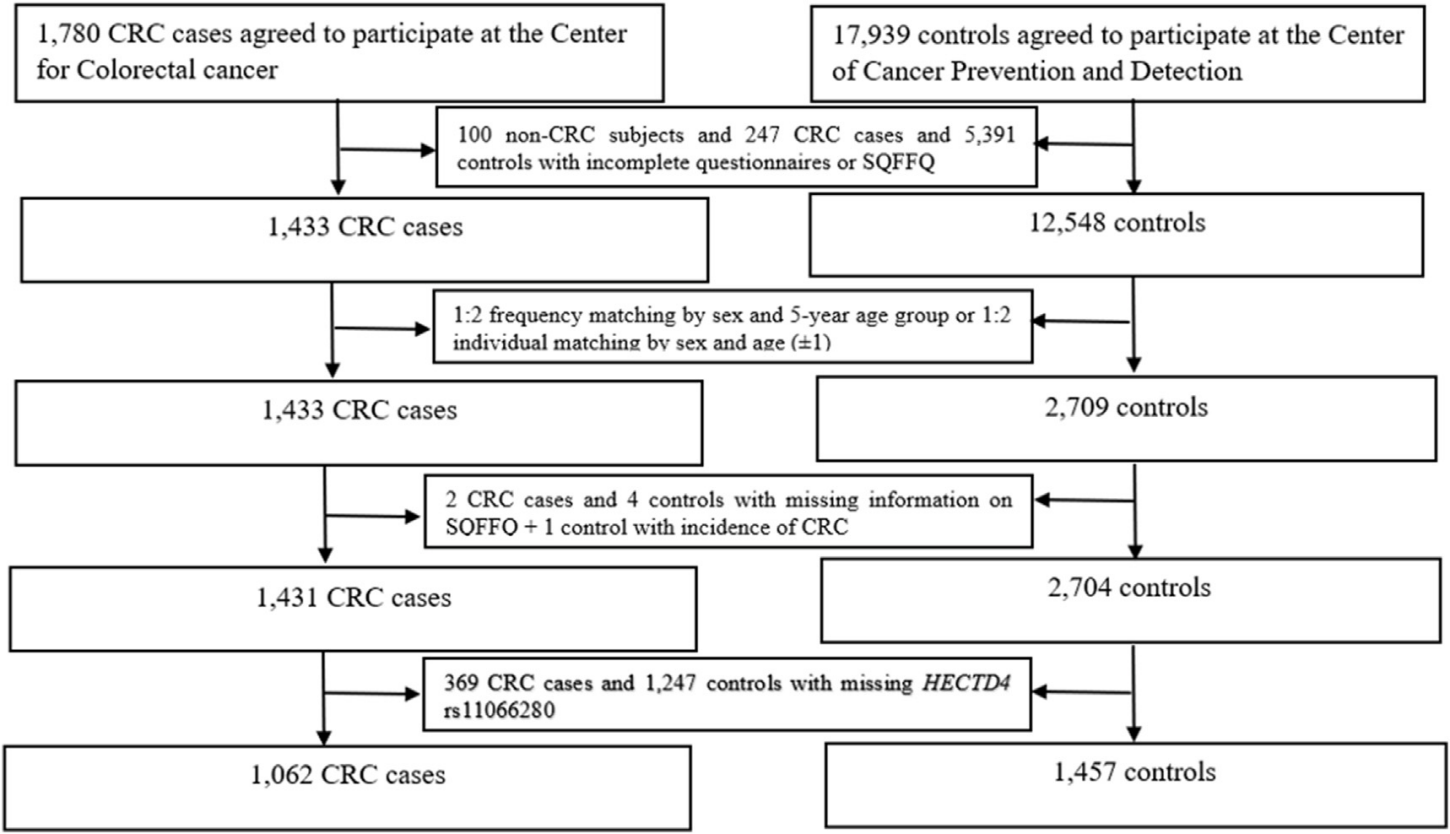
Flowchart of the study participants. A total of 1062 cases and 1457 controls were included in our final analysis after excluding participants with incomplete semiquantitative food frequency questionnaire or a self-administered questionnaire, and participants with missing information on *HECTD4* rs11066280. HECTD4, HECT domain E3 ubiquitin protein ligase 4.

We carried out a case-control study in 2010 to investigate potential dietary factors related to CRC in the Korean population. The patients with new CRC diagnosis at the Center for Colorectal Cancer of the National Cancer Center (NCC) in South Korea during 2 periods (from August 2010 to August 2013 and from January 2018 to September 2020) were selected as cases. We recruited controls from participants who visited the Center for Cancer Prevention and Detection at the NCC for their health examination from October 2007 to December 2014 and from February 2015 to June 2022. We excluded participants with incomplete semiquantitative food frequency questionnaire (SQFFQ) or a self-administered questionnaire. We matched cases and controls with 1:2 frequency matching by sex and age (±5) or 1:2 individual matching by sex and age (±1). We included 1457 controls and 1062 cases in the final analysis after excluding participants with missing information on *HECTD4* rs11066280 (Figure 1). We obtained informed consent and approval for the study protocol from all participants and the Institutional Review Board of the NCC (NCCNCS-10-350 and NCC 2015-0202), respectively.

### Data collection

A structured questionnaire was used to collect information on variables related to demography and lifestyle. Dietary intakes of participants over the previous 12 mo were obtained using the SQFFQ with 106 food items, including frequency and portion size. The SQFFQ has been reported to be valid and reliable [26]. We calculated nutrient intake using the Can-Pro 5.0 software (Computer-Aided Nutritional Analysis Program, Korea Nutrition Society).

### LCD score calculation

We equally classified participants into 11 categories based on the proportion of energy from fat, protein, and carbohydrate (9 kcal/g for fat and 4 kcal/g for protein and carbohydrate). With regard to fat and protein, individuals in the highest group earned 10 points, whereas those in the next category received 9 points, and so forth, with those in the lowest stratum receiving 0 points. The points were scored inversely for carbohydrate from 0 points for the highest group to 10 points for the lowest group. We then summed points for carbohydrate, fat, and protein to obtain an overall score, which ranges from 0 to 30 points. The high score implies a diet with low carbohydrate [9].

### Genotype measurement

Genomic DNA from the blood samples of participants was extracted using the MagAttract DNA Blood M48 Kit (Qiagen) and the BioRobot M48 automatic extraction equipment (Qiagen). The Illumina MEGA-Expanded Array (Illumina Inc.), including 123K variants was used for genotyping. The details of this method have been described previously [27]. We performed genotype imputation using the Asian population (*n* = 504) in the 1000 genomes haplotypes phase III integrated variant set release GRch37/hg19 (https://www.1000genomes.org/) as a reference panel. Genetic markers with deviation from Hardy–Weinberg equilibrium *P* values <1 × 10^–6^, a minor allele frequency <0.05, and a low call rate (<98%) were discarded. SHAPEIT (v2.r837) and IMPUTE2 (2.3.2) were used for phasing and SNP imputation, respectively. The quality control criteria were applied after filtering for an INFO score above 0.6. Finally, *HECTD4* rs11066280 was a candidate SNP for our analysis.

### Statistical analyses

The comparison of demographic, lifestyle, and dietary factors between case and control groups was performed using the chi-squared test for categorical and *t*-test for continuous variables. We used the LCD score of the control group to divide the LCD into quartiles. We used the median value for each quartile of LCD to examine the dose-response relationship of LCD score with CRC risk. We identified the association of LCD score with CRC risk by using the logistic regression models to calculate odds ratios (ORs) and 95% confidence intervals (CIs), in which we assigned the lowest quartile group as the reference group. The potential confounders were adjusted in our models, including age, sex, alcohol consumption, total energy intake, BMI, first-degree relatives with CRC, income, and smoking status. A dominant model was used for genetic association analysis. The interaction effect between LCD score and *HECTD4* rs11066280 on CRC risk was analyzed using the likelihood ratio test between the models with and without the interaction term (LCD score∗SNPs). SAS software (version 9.4, SAS Institute) was used for all statistical analyses, and a 2-sided *P* value <0.05 was considered signiﬁcant.

## Results

### General information of participants

Table 1 shows information on demographic and lifestyle factors of the subjects. The CRC cases were observed to be older compared with the healthy participants (58.4 ± 10.3 compared with 57.1 ± 9.1, *P* = 0.001). Similarly, a greater proportion of first-degree relatives with CRC was found in the cases (9.4% compared with 5.0%, *P* <0.001). Furthermore, the case group tended to have a higher proportion of ex-drinkers and individuals who did not engage in regular exercise (15.1% compared with 9.1%, *P* <0.001 and 64.1% compared with 41.9%, *P* <0.001, respectively), compared with the control group. Moreover, low levels of education, occupation, and income seemed to exhibit a higher proportion in cases than in controls (17.7% compared with 5.4%, *P* <0.001, 56.7% compared with 37.8%, *P* <0.001, and 40.7% compared with 19.6%, *P* <0.001, respectively).

**TABLE 1:** General characteristics of the subjects

	Total (*n* = 2519)			Males (*n* = 1576)			Females (*n* = 943)		
	Controls (*n* = 1457)	Cases (*n* = 1062)	*P* value^1^	Controls (*n* = 901)	Cases (*n* = 675)	*P* value^1^	Controls (*n* = 556)	Cases (*n* = 387)	*P* value^1^
Age^2^ (y)	57.1 ± 9.1	58.4 ± 10.3	0.001	57.4 ± 8.9	58.6 ± 10.1	0.010	56.6 ± 9.3	57.9 ± 10.6	0.049
BMI (kg/m^2^), *n* (%)									
<25	935 (64.2)	672 (63.3)	0.514	528 (58.6)	414 (61.3)	0.358	407 (73.2)	258 (66.7)	0.025
≥25	511 (35.1)	388 (36.5)		365 (40.5)	260 (38.5)		146 (26.3)	128 (33.1)	
Missing	11 (0.7)	2 (0.2)		8 (0.9)	1 (0.2)		3 (0.5)	1 (0.2)	
First-degree family history of CRC, *n* (%)									
No	1375 (94.4)	962 (90.6)	<0.001	856 (95.0)	611 (90.5)	<0.001	519 (93.4)	351 (90.7)	0.042
Yes	73 (5.0)	100 (9.4)		41 (4.6)	64 (9.5)		32 (5.8)	36 (9.3)	
Missing	9 (0.6)	0 (0)		4 (0.4)	0 (0)		5 (0.8)	0 (0)	
Smoking status, *n* (%)									
Nonsmoker	710 (48.7)	531 (50.0)	0.206	181 (20.1)	181 (26.8)	0.001	529 (95.1)	350 (90.4)	0.019
Ex-smoker	509 (34.9)	384 (36.2)		490 (54.4)	362 (53.6)		19 (3.4)	22 (5.7)	
Current smoker	238 (16.4)	146 (13.7)		230 (25.5)	132 (19.6)		8 (1.5)	14 (3.6)	
Missing	0 (0)	1 (0.1)		0 (0)	0 (0)		0 (0)	1 (0.3)	
Alcohol consumption, *n* (%)									
Nondrinker	470 (32.3)	415 (39.1)	<0.001	144 (16.0)	168 (24.9)	<0.001	326 (58.6)	247 (63.8)	<0.001
Ex-drinker	133 (9.1)	160 (15.1)		105 (11.7)	120 (17.8)		28 (5.0)	40 (10.3)	
Current drinker	854 (58.6)	486 (45.7)		652 (72.3)	387 (57.3)		202 (36.4)	99 (25.6)	
Missing	0 (0)	1 (0.1)		0 (0)	0 (0)		0 (0)	1 (0.3)	
Regular exercise, *n* (%)									
Yes	827 (56.8)	381 (35.9)	<0.001	535 (59.4)	247 (36.6)	<0.001	292 (52.5)	134 (34.6)	<0.001
No	610 (41.9)	681 (64.1)		353 (39.2)	428 (63.4)		257 (46.2)	253 (65.4)	
Missing	20 (1.3)	0 (0)		13 (1.4)	0 (0)		7 (1.3)	0 (0)	
Education *n* (%)									
Elementary school and lower	79 (5.4)	188 (17.7)	<0.001	38 (4.2)	87 (12.9)	<0.001	41 (7.4)	101 (26.1)	<0.001
Middle school	100 (6.9)	161 (15.2)		62 (6.9)	101 (15.0)		38 (6.8)	60 (15.5)	
High school	477 (32.7)	439 (41.3)		241 (26.8)	289 (42.8)		236 (42.5)	150 (38.8)	
College and higher	780 (53.5)	272 (25.6)		550 (61.0)	198 (29.3)		230 (41.4)	74 (19.1)	
Missing	21 (1.5)	2 (0.2)		10 (1.1)	0 (0)		11 (1.9)	2 (0.5)	
Occupation, *n* (%)									
Group 1: professional, administrative or office workers	423 (29.0)	258 (24.3)	<0.001	322 (35.7)	206 (30.5)	<0.001	101 (18.2)	52 (13.4)	<0.001
Group 2: sales or service industry workers	299 (20.5)	77 (7.3)		216 (24.0)	50 (7.4)		83 (14.9)	27 (7.0)	
Group 3: agriculturist, soldier or manufacturing workers	162 (11.1)	123 (11.6)		143 (15.9)	103 (15.3)		19 (3.4)	20 (5.2)	
Group 4: housekeeper, the jobless or others	550 (37.8)	603 (56.7)		208 (23.1)	316 (46.8)		342 (61.5)	287 (74.2)	
Missing	23 (1.6)	1 (0.1)		12 (1.3)	0 (0)		11 (2.0)	1 (0.2)	
Marital status, *n* (%)									
Married	1267 (87.0)	946 (89.1)	0.220	806 (89.5)	615 (91.1)	0.373	461 (82.9)	331 (85.5)	0.457
Others	177 (12.2)	113 (10.6)		89 (9.9)	58 (8.6)		88 (15.8)	55 (14.2)	
Missing	13 (0.8)	3 (0.3)		6 (0.6)	2 (0.3)		7 (1.3)	1 (0.3)	
Monthly income, *n* (%) (10,000 Korean Won/mo)									
<200	285 (19.6)	432 (40.7)	<0.001	171 (19.0)	274 (40.6)	<0.001	114 (20.5)	158 (40.8)	<0.001
200–400	543 (37.3)	386 (36.4)		344 (38.2)	245 (36.3)		199 (35.8)	141 (36.4)	
≥400	573 (39.3)	234 (22.0)		354 (39.3)	149 (22.1)		219 (39.4)	85 (22.0)	
Missing	56 (3.8)	10 (0.9)		32 (3.5)	7 (1.0)		24 (4.3)	3 (0.8)	
Total energy intake^2^ (kcal/d)	1793.3 ± 569.7	2082.2 ± 634.9	<0.001	1825.9 ± 544.9	2203.2 ± 605.6	<0.001	1740.5 ± 604.5	1871.1 ± 630.4	0.001
Carbohydrate^2^ (% energy/d)	69.9 ± 7.9	71.8 ± 7.3	<0.001	70.3 ± 7.5	71.5 ± 7.5	0.002	69.1 ± 8.6	72.4 ± 7.1	<0.001
Protein^2^ (% energy/d)	14.3 ± 2.6	13.4 ± 2.2	<0.001	14.1 ± 2.4	13.4 ± 2.1	<0.001	14.5 ± 2.7	13.5 ± 2.2	<0.001
Fat^2^ (% energy/d)	15.9 ± 6.0	14.8 ± 5.7	<0.001	15.6 ± 5.6	15.2 ± 5.9	0.157	16.3 ± 6.5	14.1 ± 5.4	<0.001
Low-carbohydrate diet score^2^	16.1 ± 9.0	13.5 ± 8.5	<0.001	15.7 ± 8.8	13.7 ± 8.5	<0.001	16.8 ± 9.3	13.1 ± 8.7	<0.001

1 *t*-test and χ^2^ test were used for continuous and categorical variables, respectively.

2 Mean ± SD.

### Low-carbohydrate diet score and CRC risk

In comparison with the control group, total energy intake and energy percentage from carbohydrate were more likely to be higher in the cases (2082.2 ± 634.9 compared with 1793.3 ± 569.7, *P* <0.001 and 71.8 ± 7.3 compared with 69.9 ± 7.9, *P* <0.001, respectively). In contrast, the cases tended to consume less protein and fat than the healthy participants; the percentages of energy from protein and fat were 13.4 ± 2.2 compared with 14.3 ± 2.6, *P* <0.001 and 14.8 ± 5.7 compared with 15.9 ± 6.0, *P* <0.001, respectively. Thus, the low-carbohydrate diet score was lower in those with CRC (13.5 ±8 .5 compared with 16.1 ± 9.0, *P* <0.001) (Table 1).

Table 2 represents the quartiles of LCD score in relation to CRC risk. Our results found a protective effect of high LCD score against CRC risk. In detail, in comparison with individuals in the lowest quartile group, CRC risk among participants in the highest quartile group was significantly lower. Notably, the significant association was consistent for the crude model and the adjusted model; the ORs were 0.49 (0.39–0.62) and 0.52 (0.40–0.68), *P* for trend <0.001.

**TABLE 2:** Odds ratios and 95% confidence intervals of colorectal cancer according to the quartiles of low-carbohydrate diet score

Low-carbohydrate diet score	No. of controls (%)	No. of cases (%)	Model 1	Model 2
Total (*n* = 2519)				
Q1 (<8)	330 (22.7)	307 (28.9)	1	1
Q2 (8–17)	370 (25.4)	363 (34.2)	1.06 (0.85, 1.30)	1.12 (0.89, 1.41)
Q3(17–24)	372 (25.5)	216 (20.3)	0.62 (0.50, 0.78)	0.68 (0.53, 0.87)
Q4 (≥24)	385 (26.4)	176 (16.6)	0.49 (0.39, 0.62)	0.52 (0.40, 0.68)
*P* for trend			<0.001	<0.001
Males (*n* = 1576)				
Q1 (<8)	209 (23.2)	189 (28.0)	1	1
Q2 (8–17)	228 (25.3)	227 (33.6)	1.10 (0.84, 1.44)	1.13 (0.84, 1.53)
Q3 (17–24)	237 (26.3)	141 (20.9)	0.66 (0.49, 0.88)	0.70 (0.50, 0.97)
Q4 (≥24)	227 (25.2)	118 (17.5)	0.58 (0.43, 0.77)	0.60 (0.43, 0.85)
*P* for trend			<0.001	<0.001
Females (*n* = 943)				
Q1 (<9)	132 (23.7)	130 (33.6)	1	1
Q2 (9–17)	133 (23.9)	129 (33.3)	0.99 (0.70, 1.39)	1.04 (0.72, 1.50)
Q3(17–24)	135 (24.3)	72 (18.6)	0.54 (0.37, 0.79)	0.58 (0.39, 0.87)
Q4 (≥24)	156 (28.1)	56 (14.5)	0.36 (0.25, 0.54)	0.40 (0.26, 0.62)
*P* for trend			<0.001	<0.001

Model 1: crude model.

Model 2: adjusted for age, total energy intake, BMI, first-degree family history of CRC, smoking status, alcohol consumption, and income. In the total subjects, Model 2 was additionally adjusted for sex.

Abbreviations: CRC, colorectal cancer; Q, quartile.

### Associations of *HECTD4* rs11066280 genetic polymorphisms with CRC risk in the dominant model

We used a dominant model to explore *HECTD4* rs11066280 in relation to CRC risk. There were 3 genotypes of *HECTD4* rs11066280 including TT, AA, and AT. We identified a significant association of *HECTD4* rs11066280 with CRC risk; a lower CRC risk was observed in the individuals carrying minor allele A [OR = 0.80 (0.68–0.95) in the crude model and 0.75 (0.62–0.91) in the adjusted model]. However, the genetic association seemed to be gender-specific; the protective effect was restricted to females with an OR of 0.66 (0.49–0.91) (Table 3). Furthermore, because *ALDH2* rs671 has been indicated to reduce the CRC risk; therefore; we checked the linkage disequilibrium between *HECTD4* rs11066280 and *ALDH2* rs671 and no correlation between 2 SNPs was found in our population (data not shown).

**TABLE 3:** Associations of *HECTD4* rs11066280 genetic polymorphisms with CRC risk in the dominant model

	Genotype	No. (%)		OR (95% CI)	
		Controls	Cases	Model 1	Model 2
Total	TT	960 (65.9)	750 (70.6)	1	1
	AA/AT	497 (34.1)	312 (29.4)	0.80 (0.68, 0.95)	0.75 (0.62, 0.91)
Males	TT	589 (65.4)	463 (68.6)	1	1
	AA/AT	312 (34.6)	212 (31.4)	0.86 (0.70, 1.07)	0.83 (0.65, 1.06)
Females	TT	371 (66.7)	287 (74.2)	1	1
	AA/AT	185 (33.3)	100 (25.8)	0.70 (0.52, 0.93)	0.66 (0.49, 0.91)

Model 1: crude model.

Model 2: adjusted for age, total energy intake, BMI, first-degree family history of CRC, smoking status, alcohol consumption, and income. In the total subjects, Model 2 was additionally adjusted for sex.

Abbreviations: CI, confidence interval; CRC, colorectal cancer; HECTD4, HECT domain E3 ubiquitin protein ligase 4.

### Interaction between *HECTD4* rs11066280 genetic polymorphisms and low-carbohydrate diet score with CRC risk in the dominant model

We then analyzed the association of LCD score with CRC risk stratified by genotypes to explore whether the LCD score interacts with *HECTD4* rs11066280 to modify CRC risk. In the group of participants with homozygous wildtype allele (TT), a high score of LCD served as a beneficial factor against CRC risk; the OR was 0.49 (95% CI: 0.36, 0.68) in the highest score group compared with the lowest score group. In contrast, a nonsignificant association was found for participants who carry a minor allele A. Importantly, we identified a significant interaction between LCD score and *HECTD4* rs11066280 (*P* interaction = 0.044). However, homogeneity did not emerge for males and females. The interaction only exhibited in females (*P* interaction = 0.014) (Table 4).

**TABLE 4:** Interaction between *HECTD4* rs11066280 genetic polymorphisms and low-carbohydrate diet score with CRC risk in the dominant model

	Genotype	Low-carbohydrate diet score	No. (%)		Model 1	*P* interaction	Model 2	*P* interaction
			Controls	Cases	OR (95% CI)		OR (95% CI)	
Total	TT	Q1 (<8)	214 (22.3)	238 (31.7)	1	0.037	1	0.044
		Q2 (8–17)	245 (25.5)	252 (33.6)	0.93 (0.72, 1.19)		0.97 (0.73, 1.28)	
		Q3(17–24)	245 (25.5)	138 (18.4)	0.51 (0.38, 0.67)		0.56 (0.41, 0.76)	
		Q4 (≥24)	256 (26.7)	122 (16.3)	0.43 (0.32, 0.57)		0.49 (0.36, 0.68)	
	AA/AT	Q1 (<8)	116 (23.3)	69 (22.1)	1		1	
		Q2 (8–17)	125 (25.2)	111 (35.6)	1.49 (1.01, 2.21)		1.64 (1.06, 2.54)	
		Q3(17–24)	127 (25.5)	78 (25.0)	1.03 (0.69, 1.56)		1.07 (0.68, 1.69)	
		Q4 (≥24)	129 (26.0)	54 (17.3)	0.70 (0.46, 1.09)		0.62 (0.37, 1.02)	
Males	TT	Q1 (<8)	140 (23.8)	141 (30.5)	1	0.685	1	0.662
		Q2 (8–17)	147 (25.0)	149 (32.2)	1.01 (0.73, 1.40)		1.06 (0.73, 1.53)	
		Q3(17–24)	156 (26.5)	94 (20.3)	0.60 (0.42, 0.85)		0.65 (0.44, 0.97)	
		Q4 (≥24)	146 (24.7)	79 (17.0)	0.54 (0.38, 0.77)		0.64 (0.42, 0.97)	
	AA/AT	Q1 (<8)	69 (22.0)	48 (22.6)	1		1	
		Q2 (8–17)	81 (26.0)	78 (36.8)	1.38 (0.86, 2.24)		1.47 (0.84, 2.55)	
		Q3(17–24)	81 (26.0)	47 (22.2)	0.83 (0.50, 1.40)		0.79 (0.43, 1.44)	
		Q4 (≥24)	81 (26.0)	39 (18.4)	0.69 (0.41, 1.18)		0.55 (0.29, 1.05)	
Females	TT	Q1 (<9)	78 (21.0)	107 (37.3)	1	0.009	1	0.014
		Q2 (9–17)	94 (25.3)	92 (32.1)	0.71 (0.47, 1.08)		0.73 (0.47, 1.13)	
		Q3(17–24)	91 (24.5)	47 (16.4)	0.38 (0.24, 0.60)		0.41 (0.25, 0.67)	
		Q4 (≥24)	108 (29.2)	41 (14.2)	0.28 (0.17, 0.44)		0.34 (0.20, 0.56)	
	AA/AT	Q1 (<9)	54 (29.2)	23 (23.0)	1		1	
		Q2 (9–17)	39 (21.1)	37 (37.0)	2.23 (1.15, 4.33)		2.77 (1.31, 5.85)	
		Q3(17–24)	44 (23.7)	25 (25.0)	1.33 (0.67, 2.67)		1.39 (0.65, 3.00)	
		Q4 (≥24)	48 (26.0)	15 (15.0)	0.73 (0.34, 1.57)		0.70 (0.29, 1.68)	

Model 1: crude model.

Model 2: adjusted for age, total energy intake, BMI, first-degree family history of CRC, smoking status, alcohol consumption, and income. In the total subjects, Model 2 was additionally adjusted for sex.

Abbreviations: CI, confidence interval; CRC, colorectal cancer; HECTD4, HECT domain E3 ubiquitin protein ligase 4; Q, quartile.

## Discussion

CRC risk was identified to decrease with an increased overall LCD score in a case-control study of 1457 controls and 1062 cases. In addition, we observed a significant reduction of CRC risk in variant allele carriers of *HECTD4* rs11066280. Importantly, risk of CRC may be modified by an interaction between LCD score and *HECTD4* rs11066280, with a stronger preventative effect of LCD against CRC development observed in participants who possess the homozygous wildtype allele.

To date, some studies have been conducted to explore the contribution of LCD to carcinogenesis but the effective conclusion could not be derived because of the contradictory results. A previous prospective cohort study demonstrated a positive association between overall cancer risk and LCD, whereas a protection of a high LCD score against gastric cancer was suggested [8]. In contrast, a nonsignificant association of a diet with low-carbohydrate and high protein score with cancer risk was documented in another prospective cohort study. Furthermore, this diet was indicated to be negative and positive in relation to CRC risk in females who have a high intake of saturated fat and males with high score of vegetable protein, respectively [11]. In another study, it was emphasized that replacing foods with low carbohydrate with animal-based foods was related to a higher CRC risk [28].

Notably, our finding shares a similarity with a previous study that suggested that greater adherence to LCD may be beneficial for cancer prevention regardless of the source of protein and fat [10]. There are possible mechanisms that may be proposed for this association. First, aerobic glycolysis is the main pathway for glucose metabolism in tumor cells, and both glycolysis and mitochondrial metabolisms play an important role in these cells. Although oxygen and fully functioning mitochondria are present, cancer cells exhibit significantly increased uptake of glucose and lactate production. Glucose is the main source of energy for cancer cell proliferation and division, making them glucose-dependent [10,29]. Thus, malignant behaviors may be inhibited by LCD by reducing the energy supply to cancer cells [10]. Second, insulin and insulin-like growth factor-1 are known to be involved in cancer progression [30]. Notably, a carbohydrate-restricted diet has been recognized to have favorable effects, including decreased levels of insulin, triglycerides, and blood pressure improvement [31]. As a result, this diet has been linked to the reduction of type 2 diabetes and hypo-HDL-cholesterolemia [7,32], which have been discussed as etiological factors driving an increase in CRC incidence [19,33,34]. Thus, carbohydrate restriction may also have an impact on reducing CRC risk [10,30]. Third, adiponectin level is also increased by a carbohydrate-restricted diet that contributes to promoting insulin sensitivity [35]. Furthermore, adiponectin may have a direct impact on tumor development because it plays certain roles in regulating hematopoiesis and the immune system, suppressing the growth of myelomonocyte cell lines, causing apoptosis in myelomonocytic progenitor cells, modulating gene expression associated with apoptosis in M1 cells, and reducing expression of the Bcl-2 gene [36]. Fourth, an LCD reflects a diet with high fat intake, which produces ketone bodies. It is impossible for cancer cells to convert ketone bodies to adenosine triphosphate (ATP) because of the absence of the related mitochondrial enzymes. Therefore, a diet that is high in fat may have anticancer effects [10].

Genetic polymorphisms have been indicated as potential determinants of CRC susceptibility [12]. E3 ubiquitin protein ligase is a member of the ubiquitin ligase family encoded by *HECTD4*. There are several protein and cellular functions such as cell signaling, protein trafficking, DNA repair, cell death, and cell-cycle progression, which may be modified by ubiquitination of proteins. Notably, ubiquitination of proteins involves the participation of E3 ubiquitin protein ligase in attaching ubiquitin molecules to lysine on the target protein. Thus, ubiquitin ligase plays an important role in cancer growth and metastasis. In addition, genetic variations at 12q24 were reported to be associated with cancers [15]. Our result contributes to providing insight into the association of genetic variations of *HECTD4* with CRC risk. In detail, individuals with a minor allele of rs11066280 emerged at a lower risk compared with those with a homozygous wildtype allele. Although the mechanisms underlying this association are not yet fully understood, *HECTD4* variants have potential effects on fasting glucose level. In detail, a major allele is a risk allele for diabetes, whereas the minor allele is considered as a protective allele [16,17]. In addition, a minor allele was found to significantly contribute to the reduction of triglyceride level and was associated with metabolic syndrome [16,37]. Notably, current evidence is sufficient to support the argument that participants with metabolic syndrome and its components are more sensitive to CRC, especially participants with diabetes [19,20,38,39]. It is therefore reasonable to observe a protective role of rs11066280 A-allele against CRC. However, further studies are needed to establish a direct causal relationship between *HECTD4* and CRC development.

The amount of carbohydrate determines glucose and insulin responses. Thus, LCD may be considered as a valid option for participants with obesity and type 2 diabetes [40,41]. However, an individual’s genetic background can result in different outcomes because of an interaction between genetic variants and dietary intake [25]. Thus, genetic polymorphism was hypothesized to have a modifying effect on the association of LCD with CRC in our study. We emphasized that the effect of LCD depends on individual genotypes, with a significant interaction between rs11066280 and LCD; in detail, LCD may be more important for CRC prevention in individuals carrying the rs11066280 homozygous wildtype allele (TT). The finding of our study is consistent with the fact that the T allele has been reported as a risk allele for diabetes [16]. Although the biological mechanisms are currently unclear, possible explanations may be considered. First, insulin resistance and hyperglycemia are considered underlying mechanisms that establish a causal link between diabetes and CRC [22] and may be affected by LCD. Notably, there is evidence to suggest that LCD and *HECTD4* variants can have an impact on fasting glucose levels [16,42]. Second, a protective role against CRC of high HDL-C levels has been documented [33,34], whereas hypo-HDL-cholesterolemia risk was significantly reduced as the LCD increased among the Korean population [32]. Importantly, there is an interactive effect of LCD and genetic risk score on hypo-HDL-cholesterolemia [32]. Taken together, we need to put greater emphasis on an individual’s genetic background to consider the protective effect of LCD against CRC risk.

Our study is the first conducted to explore the potential influence of LCD and *HECTD4* variants on the etiology of CRC. In addition, this is the first attempt to draw a protective effect of LCD including an interaction with genetic polymorphisms. Our findings contribute evidence to support the role of LCD in CRC prevention. However, the protective effect seems to be allele-specific. Thus, we emphasize the importance of considering the genetic characteristics of participants when establishing a diet plan for cancer prevention. Furthermore, we used a validated SQFFQ, which was designed for the Korean population to collect information on nutrient intakes. Thus, eating habits may be well-reflected in our population. In addition, sufficient information on possible confounders was collected and adjusted in our study. However, our study has some limitations. First, recall bias and selection bias may exist in our study because of the design of a case-control study. Second, the statistical power may be affected because of a limited number of participants. Third, the impact of other potential genes was not taken into account.

In conclusion, our study suggests that adherence to a carbohydrate-restricted diet may account for a reduction in CRC risk. We also provide evidence with regard to the consideration of a minor allele of *HECTD4* rs11066280 as a protective allele against CRC risk. In addition, an interactive effect of LCD and *HECTD4* rs11066280 on colorectal carcinogenesis was emphasized, which may be helpful for establishing diet plans regarding cancer prevention.

## Author contributions

The authors’ responsibilities were as follows –; TTT, MG, JL: formal analysis; TTT: preparation of original draft; MG, JK: writing—review and editing; JL, JHO, HJC, DKS, AS, JK: data curation; JL, JHO, HJC, DKS, AS: investigation; JHO, HJC, DKS, AS, JK: methodology; JK: funding acquisition, project administration, and supervision; and all authors: read and approved the final version of the manuscript.

### Conflict of interest

The authors report no conflicts of interest.

### Funding

This work was supported by grants from the National Cancer Center, Korea (2310470) and the National Research Foundation of Korea (2021R1A2C2008439).

### Data availability

Data described in the manuscript, code book, and analytic code will be made available upon request pending.
